# If Evidence is in Favor of Incisional Hernia Prevention With Mesh, why is it not Implemented?

**DOI:** 10.3389/jaws.2023.11000

**Published:** 2023-02-01

**Authors:** Breanna Durbin, Aparajita Spencer, Amanda Briese, Colston Edgerton, William W. Hope

**Affiliations:** Department of Surgery, Novant/New Hanover Medical Center, Wilmington, NC, United States

**Keywords:** prevention, hernia, mesh, incisional, implementation

## Introduction

Incisional hernias are associated with increased cost to the patient and hospital, and decreased quality of life for patients. Furthermore, the rate of hernia recurrence increases with each subsequent repair, which further compounds this cost and morbidity (1). The rate of incisional hernia requiring operative intervention in high-risk patients approaches 70%, costing the United States greater than $3 (2). billion dollars (2, 3). The true incidence of incisional hernia ranges with estimates from 2% to 50% and are due to both surgical and patient factors (4). In a study conducted from 2010 to 2014 utilizing a Nationwide Readmission Database analyzing 15, 935 patients undergoing incisional hernia repair, 19% of them were readmitted within 1 year of their index operation. Of these patients, 35% required reoperation and overall, 5% of them had recurrence of their incisional hernia and intensified the burden to patients and on the healthcare system (5). Incisional hernias develop in 13% (0%–36%) of all patients after any type of midline abdominal incision and one third (35%) will undergo subsequent repair. More-over, signs of a stabilized incidence (not an increasing incidence) in the USA were recently reported (6–8). While some risk factors for incisional hernia formation are non-modifiable, there has recently been an interest in surgical modifiable risk factors that can help decrease the incidence of incisional hernia.

One of the most important risk factors for formation of incisional hernia that the surgeon can impact relates to the closure of the abdominal incision. The two most studied factors associated with abdominal wall closure and hernia prevention relate to the suturing technique of the abdomen and the use of prophylactic mesh augmentation (PMA). There is strong evidence to support using specific suturing techniques, such as the so-called short stitch technique, as well as the use of prophylactic mesh (6). Despite well-supported evidence and recent guidelines, skepticism and a perceived lack of adoption of certain surgical techniques that could impact incisional hernia rates remain.

This paper reviews and explores some presumed reasons why hernia prevention techniques are not followed despite evidence to support their practice. Possible reasons for the lack of adoption are explored, ranging from distrust in the evidence to concern of complication, cost, and societal factors. Strategies to help improve awareness and mitigate some of these factors are also discussed, with some recommendations given on how to move this area forward in the future.

## Methods

A review of the literature including meta-analyses, randomized controlled trials, prospective cohort studies, and surveys was performed related to hernia prevention, including abdominal wall closure and prophylactic mesh, focusing on reasons why surgeons do not adhere to evidence-based practices. Secondary to paucity of published literature on this subject, expert opinions and theories based on opinion and experiences were hypothesized.

## Results

The reasons behind the lack of use of PM for IH prevention have not been well studied. We found four main reasons cited by surgeons (Table 1). The first reason is a perceived lack of evidence and literature base to support its use. While there is strong and emerging evidence to support PM in subsets of populations, the data tends to be short term and clustered to European centers. This leads surgeons to question the long-term outcomes, as well as the applicability to their practice. The second reason is concerns over financial implications of using PM. While every country has different healthcare systems and finances, the addition of mesh at an index operation often financially impacts the hospital system and surgeon, which is currently unfavorable in many instances and can lead to long-term positive financial implications being overlooked. The third reason is that surgeons seem concerned about complications associated with prophylactic procedures, especially mesh-related complications in the context of current medicolegal climates present in many countries today. Lastly, while the placement of mesh and knowledge of the abdominal wall may seem routine to hernia surgeons, many other surgeons lack the training, knowledge, and expertise to place PM, which likely contributes to its limited use.

**TABLE 1:** Review of literature with common reasons documented on reasons PMA is not used.

Study [Ref]	Type of Publication	Publication Date	Type of support (1–4)*	Summary
(1)	Systemic literature review	July 2015	Financial 2	Cost-utility analysis of Primary Suture Closure (PSC) vs. PMA for laparotomy closure demonstrates PMA to be more effective, less costly, and overall, more cost-effective than PSC
(4)	Systemic Literature Search	November 2020	Lack of knowledge/expertise (4)	Evidence supports PMA, with significant reduction in incisional hernia rate. Implementation is limited. Surgeons should be questioning why they are not using mesh reinforcement, specifically in high-risk patients
(9)	Systematic literature search	January 2022	Technique 4	Recommendations for elective midline closure technique. Guidance in selecting the optimal approach and location of abdominal wall incisions
(10)	Survey	April 2019	Technique 4	Applications of hernia prevention principles and their controversy
(15)	Prospective Cohort Study	February 2018	Complications 3	The use of PMA in colorectal surgery, when using an algorithm for patient selection, is an effective measure for prevention of IH- at the expense of other known possible complications
(18)	Multicenter double-blind randomized controlled trial	Aug 2017	Lack of Evidence 1	Randomization of 480 patients for closure: PSC, onlay or sublay. There was a significant reduction in incidence of IH with onlay mesh reinforcement- showing potential to become standard of treatment in high-risk patients
(20)	Randomized control trial	May 2021	Complications 3	PMA is not associated with increased incidence, severity, or need of infectious complications compared to PCS
(21)	Multicenter randomized control trial	April 2016	Lack of Evidence 1 Technique 4	PMA during AAA repair is safe and effective in preventing IH, with proven 2 years follow up and only added mean operative time of 16 min
(22)	Meta-analysis	June 2020	Lack of Evidence 1	PMA using onlay technique, specifically in high-risk patients, leads to significant reduction in IH

*1. Lack of evidence/literature.

2. Financial.

3. Complications.

4. Lack of training/knowledge/expertise.

## Discussion

This review highlights some of the often-cited reasons why hernia prevention principles are not practiced. Addressing these concerns will increase implementation and help facilitate these techniques becoming more widely practiced.

It is very unlikely to change surgeons’ practices if they do not believe in what they are doing or do not feel that their current practice is optimal. Disbelief and lack of awareness of current evidence are cited reasons for why surgeons have failed to embrace hernia prevention strategies. A recent survey by Fischer et al. explored reasons why surgeons did not practice current hernia prevention strategies (1). A total of 497 surgeons were included in the survey, most of whom do practice some of the recommended suturing techniques. Slowly absorbing sutures were used by 81% of respondents with 63% stating they closed using a 4:1 suture to wound (S:W) length ratio (although they did not routinely measure) and 58% stating they used the short stitch technique (although they did not routinely measure) (10, 11). Only 3% and 4% of respondents stated they have never heard of the 4:1 S:W length ratio and the short stitch technique, respectively. While these numbers relay adherence to suturing techniques, it must be remembered that this survey is likely biased and may not represent current practices in the United States and Europe, as this survey was sent to members of the European and American Hernia Society, as well as through an online Facebook group mostly comprised of hernia surgeons. It is also important to note that while the majority of surgeons stated they used a 4:1 S:W length ratio and short stitch technique, only 16% and 14%, respectively, of respondents reported measuring their ratios, which is a recommended practice (16, 10). There was less familiarity and trust of the literature for the use of PMA, with 11% of respondents stating they were unfamiliar with the literature and 23% of respondents stating they were unconvinced of the efficacy of the use of PMA.10 Despite this, it is has been proposed that high-risk patients, including those with morbid obesity, diabetes, and hypertension, could provide the most cost-effective and efficient way to target individuals that could benefit from PMA (1).

While there is evidence to support abdominal wall closures techniques and PMA, well-designed prospective randomized trials are needed. Replicating short stitch technique trials in a more diverse patient population that includes obese patients is also needed, as this patient population was not captured in many of the initial studies. Given the associated risks and concerns of PMA, this may not be appropriate for all patients, but utilizing risk calculators to identify high-risk patients who would benefit from more aggressive prevention strategies is needed. Additionally, ideal closure methods for emergent surgeries are another understudied group. Ultimately, algorithms and guidelines on when to use specific prevention strategies in specific clinical situations will be helpful in guiding and supporting surgeons.

Cost is often a barrier for new procedures and devices to overcome prior to widespread adoption. This variable can be difficult to elucidate and is frequently used to support one’s bias or opinion without performing a comprehensive cost-benefit analysis, which accounts for the long-term cost savings associated with preventative strategies. Alli V et al. used a large administrative database with over 14,000 patients to show that incisional hernia were common and increased the cost of care for individuals from 97% to 310% over 3 years (17). Gillion et al. reported the cost burden of incisional hernias in France and found that reducing the incidence of incisional hernia by 5% could result in a national cost savings of 4 million euros per year (18). Despite these data, cost is often cited as a cause for concern for lack of adoption of some hernia prevention principles. Even in comparing suture closure methods where the cost of a prosthetic material is not being considered, some surgeons argue the extra time it takes to perform a short stitch suture closure may be associated with higher operating room costs. Interestingly, the STITCH trial noted an increase of only 16 min between methods (19). The main cost concerns, however, relate to the use of prophylactic mesh as a cost-saving endeavor in hernia prevention despite good evidence to the contrary.

Time associated with the placement of PMA has also been cited as a reason why surgeons may not want to perform, although in the survey by Fischer et al. only 6% of respondents state this was the reason for not practicing (10). Studies have reported that the extra time for mesh placement ranges between ten to 20 min and is dependent on the technique performed (17–20). One way to address this barrier to adoption is to make the technique of PMA straightforward and reproducible. Onlay techniques, which have been shown to have similar efficacy in the PRIMA trial and easier and quicker fixation strategies, are being studied to help to try to improve efficiency (12).

An additional financial consideration for these techniques is reimbursement. This is further complicated by the concept of closing teams in which a surgical team will participate in the abdominal closure alone for a primary abdominal operation, such as Abdominal Aortic Aneurysm repair, which is the setting in which PMA is employed rather than during incisional hernia repair in which mesh placement is included in the primary procedure code. Whether PMA is performed by a closing team or the primary surgeon, it is important that the providers employing hernia prevention strategies are compensated for their time and expertise. A significant development for this was the approval of CPT code specifically for PMA, 0437T. This tracking code is reportedly beginning to help surgeons get reimbursed and, with additional use and outcome data, will hopefully transition to a reliable reimbursement code for performing PMA.

Related to cost, it is imperative that surgeons performing hernia prevention strategies, such as PMA, get reimbursed for their work and hopefully the tracking code will soon become a permanent code. Healthcare policymakers and insurers will also need to help ensure that ultimately what is good for the patient can be safely implemented into practice through a holistic approach to patient care.

Another often-cited reason for the lack of adoption of hernia prevention techniques is a concern for associated complications. This most often relates to the use of prophylactic mesh, but also regarding the concern that small stitch techniques may lead to abdominal dehiscence or burst abdomen, especially in the obese population. Another concern relates to the use of mesh in patients that may not have gotten a hernia and the overtreatment that would occur by using the mesh. In these patients, you subject a patient to potential mesh related complications and infectious complications for no reason, hence why risk prediction models are so important in these patients.

The use of prophylactic mesh is particularly sensitive towards today’s medical legal climate, highlighted by class action lawsuits for mesh failures. The survey by Fischer et al. saw that the most common reason for not using PMA was fear of mesh infection or mesh-related complications, cited by 46% of respondents (10). Although there is a large amount of fear related to the use of PMA, data regarding its benefits should be thoughtfully considered. The concept of “primum non-nocere: first do no harm” can be seen from both aspects of using or not using prophylactic mesh. As the data from the PRIMA trial suggests, the use of prophylactic mesh decreases risk of incisional hernia formation among high-risk patients. However, it is important to note that we do not know what risk of hernia development justifies using prophylactic mesh and therefore should be cautious in applying this concept broadly without discretion (22).

There have been two landmark randomized controlled trials (RCT) assessing incidence of incisional hernia after midline laparotomy. The PRIMA trial included 480 patients across 12 different countries undergoing elective midline laparotomy for abdominal aortic aneurysm repair or with body mass index of 27 kg/m^2^ or higher and incidence of incisional hernia formation over a two-year follow-up period. Patients were randomly assigned to one of three groups, including primary suture repair, sublay mesh repair, or onlay mesh repair. A significant reduction in the incidence of incisional hernia was achieved with onlay mesh reinforcement compared with sublay mesh reinforcement and primary suture only. There was no difference in rate of infection, re-intervention, or re-admissions between groups (12). This study suggests that PMA in an onlay fashion should be a new standard treatment for high-risk patients undergoing midline laparotomy. Van den Dop et al. further elucidated that there is no increased incidence, severity, or need for invasive treatment of infectious complications in the PRIMA trial PMA group compared to suture closure (13).

Another multicenter RCT by Muysoms et al. assessed the incidence of incisional hernia at two-year follow-up after conventional closure versus PMA with a large-pore polypropylene mesh in a retromuscular fashion for patients undergoing midline laparotomy for elective abdominal aortic aneurysm repair. There were no adverse effects seen related to PMA, apart from an increased mean time to closure of the abdominal wall for the PMA group compared with the control group. Specifically, this was 46 min compared to 30 min, and there was a significant reduction in incidence of incisional hernia from 28% in the conventional closure group to 0% in the PMA group (14). Both RCTs suggest that PMA results in decreased incidence of incisional hernia, with no difference in infectious complication rate.

Studies have shown that lack of education contributes to the low use of prophylactic mesh. In the survey by Fischer et al. 11% were unfamiliar with the literature, 24% were familiar but would still not use, 12% were unfamiliar enough with the methods to correctly execute, and 23% were unconvinced of the benefits (10).

This would suggest that education for the general surgeon population should be two-fold. First would require education about the safety and efficacy of using prophylactic mesh. Safety concerns mainly include concern for elevated surgical site infections (SSI) with the use of prophylactic mesh. 46.9% of surgeons surveyed do not use prophylactic mesh due to concern for SSI or other mesh complications.10 Systematic reviews by Depudyt et al. and Jairam et al. showed no difference in overall infection when evaluating RCTs and cohort studies (15, 4). There is also evidence indicating that prophylactic mesh has a lower rate of SSI compared to mesh that is placed for the repair of an incisional hernia.4 The second part of surgeon education would be addressing unfamiliarity with surgical techniques. This is a less common reason for not using prophylactic mesh, however it is still prevalent with 12% of surgeons reporting not being comfortable with mesh insertion (neither sublay nor onlay) (10). Although sublay mesh is known to be more physiological, it is also more technically demanding than onlay mesh repairs. The 2017 PRIMA follow-up study determined that onlay mesh and sublay mesh were equivalent in effectiveness (12). The ability to place mesh in either position may lead to more surgeons adopting the use of prophylactic mesh placement, depending on their comfort level with either procedure. In the small percentage of surgeons that are unfamiliar with either, it will be important to encourage CME, videos, and other learning opportunities to help increase surgeons’ comfort levels, so they use mesh more routinely.

Teaching and education are also important components of ensuring new techniques related to hernia prevention get implemented safely. Education and training must be available at all levels, including medical students, residents, and fellows as well as practicing surgeons with methods based on each learner’s needs. It is imperative that education is performed as a surgical community and not siloed, as many surgical subspecialties will need to be involved. To leverage expertise, partnerships with surgical societies, along with industry and surgical educators, should be established.

Lastly, and most importantly, we as surgeons must be vigilant to ensure that we care for our patients in the best way possible and take part in shared decision-making related to hernia prevention. This involves making sure we are up-to-date on new technologies, practicing evidence-based medicine, and following our outcomes. There are many groups and societies that have implemented or are in the process of implementing registries for abdominal wall closure and prophylactic mesh. These registries are important for patient safety and will help with research, including long-term outcomes.

In conclusion, there are several cited reasons why hernia prevention strategies are not implemented. While some of the reasons have validity and need attention, most are due to lack of awareness and unwarranted fear. Efforts are currently underway to help promote hernia prevention principles. These need to be expanded through the support of many stakeholders, including surgeons, industry, societies, and healthcare policymakers. Ultimately, by working together, we can make a major impact on patient care and help alleviate the burden of incisional hernias.
